# (*E*)-*N*′-(5-Bromo-2-hy­droxy­benzyl­idene)-2-(4-isobutyl­phen­yl)propano­hydrazide

**DOI:** 10.1107/S1600536812030322

**Published:** 2012-07-07

**Authors:** Shaaban K. Mohamed, Peter N. Horton, Mehmet Akkurt, Mustafa R. Albayati, Antar A. Abdelhamid

**Affiliations:** a Chemistry and Environmental Division, Manchester Metropolitan University, Manchester M1 5GD, England; b School of Chemistry, University of Southampton, Highfield, Southampton SO17 1BJ, England; c Department of Physics, Faculty of Sciences, Erciyes University, 38039 Kayseri, Turkey; d Kirkuk University, College of Science, Department of chemistry, Kirkuk, Iraq

## Abstract

The title compound, C_20_H_23_BrN_2_O_2_, containing an ibuprofen core, crystallizes with three independent mol­ecules of similar conformation in the asymmetric unit. In these three mol­ecules, the two benzene rings make dihedral angles of 82.7 (2), 71.2 (2) and 78.0 (3)° with respect to each other. The atoms of the isobutyl groups in two of the mol­ecules are disordered over two positions, with site-occupancy ratios of 0.516 (8):0.484 (8) and 0.580 (8):0.420 (8). In the crystal, mol­ecules are linked by N—H⋯O, C—H⋯O and O—H⋯N hydrogen bonds. Furthermore, C—H⋯π inter­actions are also observed.

## Related literature

For pharmaceutical applications of ibuprofen, see: Cohen & Harris (1987 ▶); Palaska *et al.* (2002 ▶); Aktay *et al.* (2005 ▶); Bedia *et al.* (2006 ▶); Rollas *et al.* (2002 ▶); Terzioglu & Gürsoy (2003 ▶). For the synthesis of potential biologically active compounds incorporating the ibuprofen sub-structure, see: Mohamed *et al.* (2012 ▶); Amir & Kumar (2005 ▶). For related structures, see: Goh *et al.* (2010 ▶); Fun *et al.* (2009*a* ▶,*b* ▶); Wu *et al.* (2010 ▶).
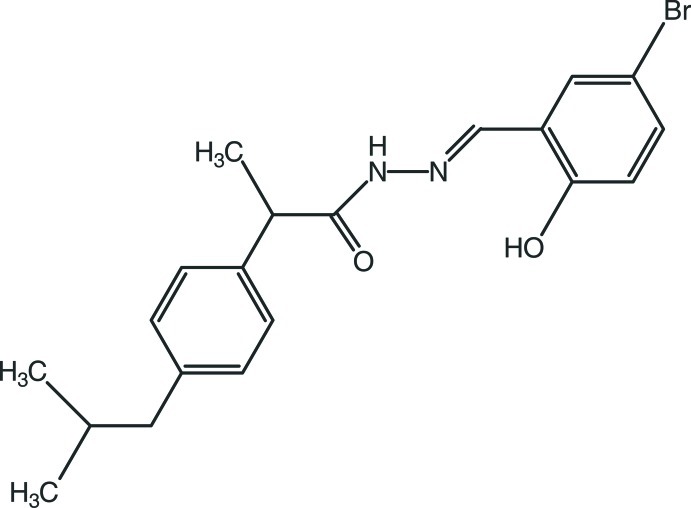


## Experimental

### 

#### Crystal data

C_20_H_23_BrN_2_O_2_
*M* *_r_* = 403.30
Monoclinic, 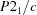

*a* = 16.598 (8) Å
*b* = 35.455 (18) Å
*c* = 9.821 (5) Å
β = 90.718 (5)°
*V* = 5779 (5) Å^3^
*Z* = 12
Mo *K*α radiation
μ = 2.15 mm^−1^
*T* = 100 K
0.24 × 0.05 × 0.04 mm

#### Data collection

Rigaku Saturn724+ diffractometer
Absorption correction: multi-scan (*CrystalClear*; Rigaku, 2001 ▶) *T*_min_ = 0.879, *T*_max_ = 0.918
52355 measured reflections
11610 independent reflections
9142 reflections with *I* > 2σ(*I*)
*R*_int_ = 0.054

#### Refinement

*R*[*F* ^2^ > 2σ(*F* ^2^)] = 0.089
*wR*(*F* ^2^) = 0.246
*S* = 1.11
11610 reflections
748 parameters
206 restraints
H atoms treated by a mixture of independent and constrained refinement
Δρ_max_ = 1.05 e Å^−3^
Δρ_min_ = −2.39 e Å^−3^

### 

Data collection: *CrystalClear* (Rigaku, 2001 ▶); cell refinement: *CrystalClear*; data reduction: *CrystalClear*; program(s) used to solve structure: *SHELXS97* (Sheldrick, 2008 ▶); program(s) used to refine structure: *SHELXL97* (Sheldrick, 2008 ▶); molecular graphics: *ORTEP-3 for Windows* (Farrugia, 1997 ▶); software used to prepare material for publication: *WinGX* (Farrugia, 1999 ▶) and *PLATON* (Spek, 2009 ▶).

## Supplementary Material

Crystal structure: contains datablock(s) global, I. DOI: 10.1107/S1600536812030322/im2391sup1.cif

Structure factors: contains datablock(s) I. DOI: 10.1107/S1600536812030322/im2391Isup2.hkl

Supplementary material file. DOI: 10.1107/S1600536812030322/im2391Isup3.cml

Additional supplementary materials:  crystallographic information; 3D view; checkCIF report

## Figures and Tables

**Table 1:** Hydrogen-bond geometry (Å, °) *Cg*2, *Cg*3, *Cg*4 and *Cg*6 are the centroids of the C31–C36, C41–C46, C51–C56 and C11–C16 benzene rings, respectively.

*D*—H⋯*A*	*D*—H	H⋯*A*	*D*⋯*A*	*D*—H⋯*A*
O1—H1⋯N1	0.84	1.90	2.626 (5)	143
N2—H2*A*⋯O2^i^	0.88 (2)	1.98 (2)	2.804 (5)	156 (4)
O21—H21⋯N21	0.84	1.89	2.616 (5)	144
N22—H22*A*⋯O22^i^	0.88 (2)	1.98 (2)	2.803 (5)	155 (4)
O41—H41⋯N41	0.84	1.87	2.610 (5)	146
N42—H42*A*⋯O42^i^	0.88 (2)	1.98 (1)	2.813 (5)	158 (4)
C27—H27⋯O22^i^	0.95	2.55	3.218 (6)	128
C55—H55⋯O41^i^	0.95	2.55	3.357 (6)	142
C10—H10*A*⋯*Cg*4	0.98	2.78	3.734 (6)	164
C30—H30*B*⋯*Cg*6^ii^	0.98	2.72	3.674 (6)	163
C50—H50*A*⋯*Cg*2	0.98	2.78	3.733 (5)	163
C59*A*—H59*C*⋯*Cg*3^i^	0.98	2.94	3.824 (12)	151

Symmetry codes: (i) 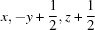; (ii) 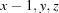.

**Fig. 1.**
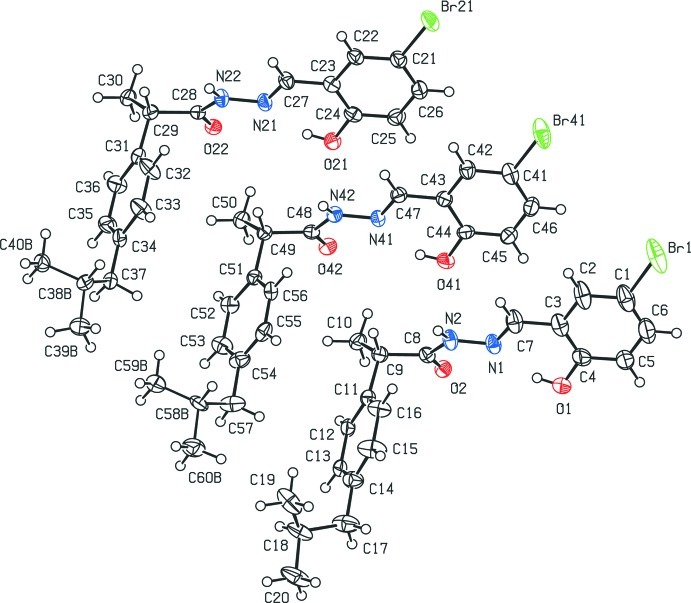
View of the title compound showing displacement ellipsoids for non-H atoms at the 50% probability level. Only the major disorder components are shown.

**Fig. 2.**
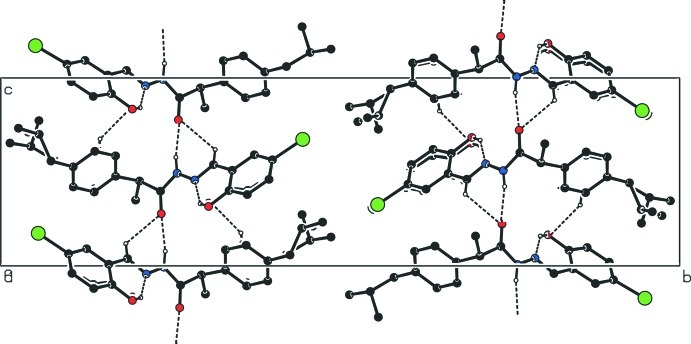
View of the molecular packing and the hydrogen bonding along the a axis. The minor disorder components and hydrogen atoms not involved in hydrogen bonding have been omitted for clarity.

## supplementary crystallographic information

### Comment

Compounds incorporating the ibuprofen core are of great interest for chemists and biologists due to their significant bioactivities such as anti-inflammatory, analgesic, anti-microbial and anti-tumor activities (Cohen & Harris, 1987; Palaska *et al.*, 2002; Aktay *et al.*, 2005; Bedia *et al.*, 2006; Rollas *et al.*, 2002; Terzioglu & Gürsoy, 2003). In particular, hydrazones incoporating the ibuprofen nucleus have demonstrated a variety of pharmacological activities (Bedia *et al.*, 2006; Rollas *et al.*, 2002; Terzioglu & Gürsoy, 2003). In light of such observations and following our ongoing study of non-steroidal anti-inflammatory drugs (NSAID), we are herein reporting the structure and synthesis of the title compound (I).

As shown in Fig. 1, the asymmetric unit of (I) contains three independent molecules of similar conformation and the same orientations. Their bond lengths and bond angles are in normal range and are similar to each other and those reported for related structures (Mohamed *et al.*, 2012; Amir & Kumar, 2005; Goh *et al.*, 2010; Fun *et al.*, 2009*a*,*b*; Wu *et al.*, 2010). In these three molecules A (with Br21), B (with Br41) and C (with Br1), the dihedral angles between the two benzene rings are 82.7 (2), 71.2 (2) and 78.0 (3)°, respectively.

The crystal packing is stabilized by C—H···O, N—H···O and O—H···N hydrogen bonds (Table 1, Fig. 2), forming a three dimensional network. In addition, C—H···π interactions (Table 1) help to stabilize the crystal structure.

### Experimental

A mixture of 220 mg (1 mmol) 2-(4-isobutylphenyl)propane hydrazide and 201 mg (1 mmol) 5-bromo-2-hydroxybenzaldehyde was grinded in a mortar with a pestle before being well mixed with three drops of acetic acid. The powder mixture was transferred and homogeneously spread in a Petri dish, and then irradiated with microwaves (at 600 W) for a total of 2 min at intervals of 30 s. The resulting product was collected and crystallized from ethanol to obtain prismatic crystals in 92% yield with a m.p. of 434–436 K.

### Refinement

In the asymmetric unit, the atoms (C38, C39 and C40 for molecule A, and C58, C59 and C60 for molecule B) of the isobutyl groups of two of the three molecules are disordered over two positions, with site-occupancy ratios of 0.516 (8):0.484 (8) and 0.580 (8):0.420 (8), respectively. Both geometrical (SADI and DFIX) and displacement (SIMU) restraints were employed on these atoms. H atoms bound to C atoms and the hydroxyl H atoms were placed in calculated positions [O—H = 0.84 Å and C—H = 0.95 - 1.00 Å] and were refined by using a riding model approximation, with *U*_iso_(H) = 1.2 or 1.5*U*_eq_(C, O). The NH hydrogen atoms are localized from difference Fourier maps [N2—H2A = 0.88 (2) Å, N22—H22A = 0.88 (2) Å and N42—H42A = 0.882 (18) Å] and were also refined by using a riding model approximation, with *U*_iso_(H) = 1.2*U*_eq_(N). The highest peak is 0.86 Å from Br1 and the deepest hole is 0.81 Å from Br21.

### Crystal data

**Table:**

C_20_H_23_BrN_2_O_2_	*F*(000) = 2496
*M**_r_* = 403.30	*D*_x_ = 1.391 Mg m^−^^3^
Monoclinic, *P*2_1_/*c*	Mo *K*α radiation, λ = 0.71075 Å
Hall symbol: -P 2ybc	Cell parameters from 14415 reflections
*a* = 16.598 (8) Å	θ = 2.3–30.7°
*b* = 35.455 (18) Å	µ = 2.15 mm^−^^1^
*c* = 9.821 (5) Å	*T* = 100 K
β = 90.718 (5)°	Slab, colourless
*V* = 5779 (5) Å^3^	0.24 × 0.05 × 0.04 mm
*Z* = 12	

### Data collection

**Table:**

Rigaku Saturn724+ diffractometer	11610 independent reflections
Radiation source: Rotating anode	9142 reflections with *I* > 2σ(*I*)
Confocal monochromator	*R*_int_ = 0.054
Detector resolution: 28.5714 pixels mm^-1^	θ_max_ = 26.5°, θ_min_ = 3.0°
profile data from ω scans	*h* = −20→20
Absorption correction: multi-scan (*CrystalClear*; Rigaku, 2001)	*k* = −44→44
*T*_min_ = 0.879, *T*_max_ = 0.918	*l* = −12→11
52355 measured reflections	

### Refinement

**Table:**

Refinement on *F*^2^	Secondary atom site location: difference Fourier map
Least-squares matrix: full	Hydrogen site location: inferred from neighbouring sites
*R*[*F*^2^ > 2σ(*F*^2^)] = 0.089	H atoms treated by a mixture of independent and constrained refinement
*wR*(*F*^2^) = 0.246	*w* = 1/[σ^2^(*F*_o_^2^) + (0.1019*P*)^2^ + 11.0037*P*] where *P* = (*F*_o_^2^ + 2*F*_c_^2^)/3
*S* = 1.11	(Δ/σ)_max_ < 0.001
11610 reflections	Δρ_max_ = 1.05 e Å^−^^3^
748 parameters	Δρ_min_ = −2.39 e Å^−^^3^
206 restraints	Extinction correction: *SHELXL97* (Sheldrick, 2008), Fc* = kFc[1+0.001Fc^2^λ^3^/sin(2θ)]^-1/4^
Primary atom site location: structure-invariant direct methods	Extinction coefficient: 0.0026 (3)

### Special details

**Table:**

Geometry. Bond distances, angles *etc*. have been calculated using the rounded fractional coordinates. All su's are estimated from the variances of the (full) variance-covariance matrix. The cell e.s.d.'s are taken into account in the estimation of distances, angles and torsion angles
Refinement. Refinement on *F*^2^ for ALL reflections except those flagged by the user for potential systematic errors. Weighted *R*-factors *wR* and all goodnesses of fit *S* are based on *F*^2^, conventional *R*-factors *R* are based on *F*, with *F* set to zero for negative *F*^2^. The observed criterion of *F*^2^ > σ(*F*^2^) is used only for calculating -*R*-factor-obs *etc*. and is not relevant to the choice of reflections for refinement. *R*-factors based on *F*^2^ are statistically about twice as large as those based on *F*, and *R*-factors based on ALL data will be even larger.

### Fractional atomic coordinates and isotropic or equivalent isotropic displacement parameters (Å^2^)

**Table:**

	*x*	*y*	*z*	*U*_iso_*/*U*_eq_	Occ. (<1)
Br21	0.12220 (3)	0.44480 (1)	0.67351 (6)	0.0435 (2)	
O21	0.10230 (19)	0.30610 (8)	0.3163 (3)	0.0298 (10)	
O22	−0.08833 (18)	0.23640 (8)	0.2778 (3)	0.0287 (10)	
N21	−0.0232 (2)	0.28565 (10)	0.4569 (4)	0.0291 (11)	
N22	−0.0819 (2)	0.25980 (10)	0.4931 (4)	0.0270 (11)	
C21	0.1163 (3)	0.40014 (12)	0.5648 (5)	0.0307 (14)	
C22	0.0579 (3)	0.37356 (11)	0.5913 (5)	0.0288 (14)	
C23	0.0516 (3)	0.34093 (11)	0.5091 (5)	0.0267 (14)	
C24	0.1058 (3)	0.33634 (11)	0.4009 (5)	0.0274 (14)	
C25	0.1656 (3)	0.36308 (13)	0.3798 (5)	0.0330 (14)	
C26	0.1709 (3)	0.39512 (12)	0.4603 (5)	0.0324 (16)	
C27	−0.0107 (3)	0.31331 (12)	0.5387 (5)	0.0293 (16)	
C28	−0.1099 (2)	0.23528 (11)	0.3976 (5)	0.0226 (14)	
C29	−0.1658 (3)	0.20436 (12)	0.4520 (5)	0.0276 (14)	
C30	−0.2352 (3)	0.19854 (12)	0.3502 (5)	0.0344 (14)	
C31	−0.1140 (3)	0.16919 (12)	0.4768 (5)	0.0276 (14)	
C32	−0.0604 (3)	0.16906 (12)	0.5856 (5)	0.0391 (16)	
C33	−0.0097 (3)	0.13855 (13)	0.6100 (6)	0.0432 (18)	
C34	−0.0116 (3)	0.10662 (12)	0.5271 (5)	0.0284 (14)	
C35	−0.0662 (3)	0.10676 (12)	0.4199 (6)	0.0364 (16)	
C36	−0.1164 (3)	0.13770 (13)	0.3935 (5)	0.0351 (16)	
C37	0.0436 (3)	0.07391 (12)	0.5580 (5)	0.0323 (14)	
C38B	0.0359 (5)	0.0566 (2)	0.7002 (9)	0.030 (2)	0.516 (8)
C39B	0.0989 (6)	0.0260 (3)	0.7328 (11)	0.037 (3)	0.516 (8)
C40B	−0.0496 (5)	0.0403 (3)	0.7117 (12)	0.033 (3)	0.516 (8)
C40A	−0.0359 (6)	0.0545 (3)	0.7730 (11)	0.029 (3)	0.484 (8)
C38A	0.0021 (5)	0.0414 (2)	0.6429 (9)	0.023 (2)	0.484 (8)
C39A	0.0658 (5)	0.0112 (2)	0.6694 (10)	0.026 (3)	0.484 (8)
Br41	0.44109 (5)	0.44893 (2)	0.68524 (6)	0.0601 (3)	
O41	0.4368 (2)	0.30688 (9)	0.3470 (4)	0.0403 (11)	
O42	0.24456 (19)	0.23801 (8)	0.2882 (3)	0.0280 (10)	
N41	0.3033 (2)	0.28849 (9)	0.4680 (4)	0.0253 (11)	
N42	0.2440 (2)	0.26253 (10)	0.5018 (4)	0.0250 (11)	
C41	0.4393 (3)	0.40321 (13)	0.5840 (5)	0.0374 (16)	
C42	0.3779 (3)	0.37751 (12)	0.6016 (5)	0.0313 (16)	
C43	0.3753 (3)	0.34426 (12)	0.5238 (5)	0.0273 (14)	
C44	0.4363 (3)	0.33784 (12)	0.4274 (5)	0.0316 (14)	
C45	0.4982 (3)	0.36399 (13)	0.4135 (6)	0.0393 (18)	
C46	0.5000 (3)	0.39663 (13)	0.4910 (6)	0.0380 (16)	
C47	0.3112 (3)	0.31681 (11)	0.5482 (5)	0.0280 (14)	
C48	0.2216 (2)	0.23641 (11)	0.4067 (4)	0.0233 (11)	
C49	0.1677 (2)	0.20507 (11)	0.4602 (5)	0.0244 (11)	
C50	0.0997 (3)	0.19782 (12)	0.3567 (5)	0.0327 (14)	
C51	0.2232 (3)	0.17083 (11)	0.4861 (5)	0.0256 (14)	
C52	0.2170 (3)	0.13776 (12)	0.4111 (5)	0.0310 (16)	
C53	0.2700 (3)	0.10772 (13)	0.4361 (5)	0.0376 (16)	
C54	0.3317 (3)	0.11032 (14)	0.5330 (5)	0.0363 (17)	
C55	0.3365 (3)	0.14371 (13)	0.6076 (5)	0.0330 (16)	
C56	0.2831 (3)	0.17339 (12)	0.5861 (5)	0.0290 (14)	
C57	0.3903 (3)	0.07850 (16)	0.5587 (6)	0.0473 (17)	
C58B	0.3678 (5)	0.0570 (2)	0.6982 (11)	0.037 (2)	0.580 (8)
C59B	0.2825 (5)	0.0407 (2)	0.7121 (10)	0.040 (3)	0.580 (8)
C60B	0.4306 (6)	0.0261 (3)	0.7307 (12)	0.047 (3)	0.580 (8)
C60A	0.4291 (6)	0.0162 (3)	0.6349 (15)	0.035 (4)	0.420 (8)
C58A	0.3637 (6)	0.0455 (3)	0.6409 (11)	0.031 (3)	0.420 (8)
C59A	0.3349 (7)	0.0567 (3)	0.7758 (12)	0.037 (3)	0.420 (8)
Br1	0.77454 (5)	0.44894 (2)	0.67199 (7)	0.0707 (3)	
O1	0.7758 (2)	0.30621 (10)	0.3347 (5)	0.0574 (16)	
O2	0.57913 (18)	0.23839 (8)	0.2775 (3)	0.0290 (10)	
N1	0.6427 (2)	0.28700 (10)	0.4587 (4)	0.0322 (14)	
N2	0.5836 (2)	0.26125 (10)	0.4922 (4)	0.0310 (11)	
C1	0.7770 (4)	0.40254 (15)	0.5740 (7)	0.052 (2)	
C2	0.7170 (4)	0.37598 (14)	0.5938 (6)	0.0478 (19)	
C3	0.7158 (3)	0.34256 (13)	0.5156 (6)	0.0385 (16)	
C4	0.7757 (3)	0.33707 (13)	0.4165 (7)	0.047 (2)	
C5	0.8363 (3)	0.36378 (15)	0.4021 (8)	0.064 (3)	
C6	0.8373 (4)	0.39631 (16)	0.4801 (8)	0.067 (3)	
C7	0.6528 (3)	0.31489 (14)	0.5406 (6)	0.0414 (17)	
C8	0.5571 (3)	0.23665 (11)	0.3958 (5)	0.0257 (14)	
C9	0.5005 (3)	0.20593 (12)	0.4476 (5)	0.0286 (14)	
C10	0.4334 (3)	0.19931 (13)	0.3409 (5)	0.0334 (14)	
C11	0.5533 (3)	0.17124 (12)	0.4790 (5)	0.0283 (14)	
C12	0.5549 (3)	0.13965 (12)	0.3948 (5)	0.0304 (14)	
C13	0.6077 (3)	0.10950 (12)	0.4227 (5)	0.0332 (14)	
C14	0.6619 (3)	0.11110 (13)	0.5322 (5)	0.0373 (16)	
C15	0.6575 (4)	0.14264 (16)	0.6173 (6)	0.0504 (19)	
C16	0.6044 (3)	0.17217 (14)	0.5919 (5)	0.0377 (17)	
C17	0.7208 (4)	0.07928 (16)	0.5611 (6)	0.053 (2)	
C18	0.6820 (4)	0.04430 (13)	0.6358 (5)	0.0418 (18)	
C19	0.6476 (4)	0.05617 (14)	0.7730 (5)	0.056 (2)	
C20	0.7453 (4)	0.01358 (15)	0.6537 (7)	0.061 (2)	
H21	0.06000	0.29380	0.33100	0.0450*	
H26	0.21140	0.41340	0.44410	0.0390*	
H27	−0.04210	0.31580	0.61850	0.0360*	
H29	−0.18840	0.21290	0.54080	0.0330*	
H30A	−0.26550	0.22210	0.34000	0.0520*	
H22	0.02190	0.37720	0.66470	0.0350*	
H22A	−0.098 (3)	0.2578 (14)	0.5776 (17)	0.0320*	
H25	0.20340	0.35930	0.30920	0.0400*	
H33	0.02710	0.13950	0.68480	0.0520*	
H35	−0.06970	0.08530	0.36240	0.0430*	
H36	−0.15260	0.13710	0.31780	0.0420*	
H37C	0.09990	0.08240	0.54620	0.0380*	0.516 (8)
H37D	0.03300	0.05390	0.48990	0.0380*	0.516 (8)
H38B	0.04210	0.07730	0.76890	0.0370*	0.516 (8)
H39D	0.12960	0.02030	0.65070	0.0560*	0.516 (8)
H39E	0.07160	0.00310	0.76400	0.0560*	0.516 (8)
H39F	0.13570	0.03510	0.80440	0.0560*	0.516 (8)
H40D	−0.05610	0.02900	0.80200	0.0500*	0.516 (8)
H40E	−0.05790	0.02100	0.64160	0.0500*	0.516 (8)
H40F	−0.08920	0.06050	0.69900	0.0500*	0.516 (8)
H30B	−0.27100	0.17870	0.38350	0.0520*	
H30C	−0.21350	0.19110	0.26180	0.0520*	
H32	−0.05810	0.19020	0.64480	0.0470*	
H37A	0.09110	0.08330	0.60960	0.0380*	0.484 (8)
H37B	0.06290	0.06320	0.47120	0.0380*	0.484 (8)
H38A	−0.04120	0.03000	0.58440	0.0280*	0.484 (8)
H39A	0.05670	−0.00040	0.75840	0.0390*	0.484 (8)
H39B	0.11950	0.02270	0.66860	0.0390*	0.484 (8)
H39C	0.06220	−0.00810	0.59830	0.0390*	0.484 (8)
H40A	−0.05820	0.03280	0.82140	0.0430*	0.484 (8)
H40B	−0.07920	0.07250	0.75170	0.0430*	0.484 (8)
H40C	0.00490	0.06690	0.83060	0.0430*	0.484 (8)
H41	0.39570	0.29380	0.36220	0.0600*	
H46	0.54240	0.41440	0.48050	0.0450*	
H47	0.27580	0.32000	0.62260	0.0340*	
H49	0.14390	0.21330	0.54850	0.0290*	
H50A	0.06480	0.17760	0.39030	0.0490*	
H42	0.33730	0.38230	0.66660	0.0380*	
H42A	0.231 (3)	0.2602 (13)	0.5882 (13)	0.0300*	
H45	0.53970	0.35940	0.35000	0.0470*	
H53	0.26370	0.08500	0.38570	0.0450*	
H55	0.37760	0.14630	0.67530	0.0390*	
H56	0.28750	0.19550	0.64000	0.0350*	
H57C	0.44610	0.08840	0.56230	0.0570*	0.580 (8)
H57D	0.38680	0.05960	0.48460	0.0570*	0.580 (8)
H58B	0.37370	0.07620	0.77230	0.0450*	0.580 (8)
H59D	0.27450	0.02040	0.64540	0.0600*	0.580 (8)
H59E	0.24270	0.06060	0.69560	0.0600*	0.580 (8)
H59F	0.27590	0.03060	0.80420	0.0600*	0.580 (8)
H60D	0.42000	0.01540	0.82060	0.0700*	0.580 (8)
H60E	0.48470	0.03720	0.73030	0.0700*	0.580 (8)
H60F	0.42700	0.00620	0.66170	0.0700*	0.580 (8)
H50B	0.12270	0.19030	0.26950	0.0490*	
H50C	0.06800	0.22090	0.34420	0.0490*	
H52	0.17660	0.13550	0.34230	0.0370*	
H57A	0.40770	0.06890	0.46910	0.0570*	0.420 (8)
H57B	0.43860	0.08930	0.60430	0.0570*	0.420 (8)
H58A	0.31610	0.03450	0.59150	0.0370*	0.420 (8)
H59A	0.31780	0.03430	0.82600	0.0550*	0.420 (8)
H59B	0.28920	0.07400	0.76510	0.0550*	0.420 (8)
H59C	0.37850	0.06930	0.82620	0.0550*	0.420 (8)
H60A	0.41150	−0.00670	0.68210	0.0530*	0.420 (8)
H60B	0.47800	0.02600	0.67920	0.0530*	0.420 (8)
H60C	0.44030	0.01020	0.53960	0.0530*	0.420 (8)
H1	0.73160	0.29480	0.34140	0.0860*	
H2	0.67680	0.38020	0.66000	0.0570*	
H2A	0.568 (3)	0.2589 (14)	0.5769 (18)	0.0370*	
H5	0.87770	0.35970	0.33780	0.0770*	
H6	0.87920	0.41430	0.46930	0.0800*	
H7	0.61920	0.31750	0.61750	0.0500*	
H9	0.47550	0.21480	0.53400	0.0340*	
H10A	0.39680	0.17970	0.37340	0.0500*	
H10B	0.45730	0.19130	0.25500	0.0500*	
H10C	0.40330	0.22280	0.32640	0.0500*	
H12	0.52000	0.13850	0.31760	0.0360*	
H13	0.60640	0.08780	0.36620	0.0400*	
H15	0.69200	0.14390	0.69510	0.0600*	
H16	0.60310	0.19310	0.65210	0.0450*	
H17A	0.76570	0.08910	0.61810	0.0630*	
H17B	0.74370	0.07070	0.47390	0.0630*	
H18	0.63710	0.03420	0.57750	0.0500*	
H19A	0.60770	0.07620	0.75890	0.0840*	
H19B	0.69130	0.06550	0.83210	0.0840*	
H19C	0.62190	0.03440	0.81610	0.0840*	
H20A	0.72130	−0.00820	0.69910	0.0910*	
H20B	0.79020	0.02320	0.70920	0.0910*	
H20C	0.76520	0.00590	0.56420	0.0910*	

### Atomic displacement parameters (Å^2^)

**Table:**

	*U*^11^	*U*^22^	*U*^33^	*U*^12^	*U*^13^	*U*^23^
Br21	0.0529 (4)	0.0247 (3)	0.0529 (4)	−0.0068 (2)	0.0004 (3)	−0.0071 (2)
O21	0.0304 (18)	0.0298 (15)	0.0292 (19)	−0.0008 (12)	−0.0006 (14)	−0.0009 (13)
O22	0.0289 (17)	0.0263 (15)	0.031 (2)	0.0012 (12)	0.0022 (14)	−0.0040 (13)
N21	0.031 (2)	0.0242 (17)	0.032 (2)	−0.0035 (14)	−0.0003 (17)	0.0062 (15)
N22	0.032 (2)	0.0260 (17)	0.023 (2)	−0.0068 (14)	0.0014 (17)	0.0015 (15)
C21	0.031 (2)	0.026 (2)	0.035 (3)	−0.0014 (17)	−0.004 (2)	−0.0013 (19)
C22	0.032 (2)	0.0233 (19)	0.031 (3)	−0.0006 (16)	−0.003 (2)	−0.0037 (18)
C23	0.032 (2)	0.026 (2)	0.022 (3)	0.0017 (17)	−0.0002 (19)	0.0002 (17)
C24	0.031 (2)	0.025 (2)	0.026 (3)	0.0049 (16)	−0.0078 (19)	−0.0003 (17)
C25	0.027 (2)	0.036 (2)	0.036 (3)	0.0030 (18)	−0.001 (2)	0.002 (2)
C26	0.032 (3)	0.029 (2)	0.036 (3)	−0.0036 (17)	−0.004 (2)	0.005 (2)
C27	0.036 (3)	0.025 (2)	0.027 (3)	−0.0039 (17)	0.002 (2)	−0.0020 (18)
C28	0.022 (2)	0.0217 (19)	0.024 (3)	0.0024 (15)	0.0005 (18)	−0.0004 (16)
C29	0.025 (2)	0.027 (2)	0.031 (3)	0.0002 (16)	0.0035 (19)	−0.0030 (18)
C30	0.021 (2)	0.031 (2)	0.051 (3)	−0.0033 (17)	−0.001 (2)	−0.007 (2)
C31	0.024 (2)	0.028 (2)	0.031 (3)	−0.0024 (16)	0.0076 (19)	0.0026 (18)
C32	0.062 (3)	0.023 (2)	0.032 (3)	0.001 (2)	−0.016 (3)	−0.0014 (19)
C33	0.058 (4)	0.029 (2)	0.042 (3)	0.001 (2)	−0.023 (3)	−0.002 (2)
C34	0.028 (2)	0.025 (2)	0.032 (3)	−0.0077 (16)	−0.0030 (19)	0.0017 (18)
C35	0.036 (3)	0.028 (2)	0.045 (3)	−0.0009 (18)	−0.003 (2)	−0.013 (2)
C36	0.034 (3)	0.032 (2)	0.039 (3)	0.0021 (18)	−0.014 (2)	−0.009 (2)
C37	0.025 (2)	0.027 (2)	0.045 (3)	−0.0027 (16)	0.0042 (19)	0.0062 (19)
C38B	0.027 (4)	0.032 (4)	0.032 (4)	0.001 (3)	0.008 (3)	−0.002 (3)
C39B	0.037 (5)	0.037 (5)	0.038 (6)	0.007 (4)	−0.003 (4)	0.002 (4)
C40B	0.030 (4)	0.029 (5)	0.040 (6)	−0.001 (3)	−0.004 (4)	0.009 (4)
C40A	0.020 (4)	0.033 (5)	0.034 (6)	−0.002 (4)	0.003 (4)	0.000 (4)
C38A	0.013 (4)	0.027 (4)	0.030 (4)	−0.006 (3)	0.002 (3)	0.007 (3)
C39A	0.026 (5)	0.026 (4)	0.027 (5)	0.000 (3)	0.008 (4)	0.009 (4)
Br41	0.0961 (6)	0.0448 (3)	0.0398 (4)	−0.0357 (3)	0.0117 (3)	−0.0124 (2)
O41	0.043 (2)	0.0264 (16)	0.052 (2)	0.0028 (13)	0.0212 (18)	−0.0027 (15)
O42	0.0310 (17)	0.0346 (16)	0.0185 (17)	0.0012 (12)	0.0027 (13)	−0.0013 (13)
N41	0.0262 (19)	0.0227 (17)	0.027 (2)	−0.0030 (13)	−0.0015 (15)	0.0020 (14)
N42	0.030 (2)	0.0272 (17)	0.018 (2)	−0.0015 (14)	0.0054 (16)	−0.0017 (15)
C41	0.047 (3)	0.030 (2)	0.035 (3)	−0.008 (2)	−0.002 (2)	0.004 (2)
C42	0.036 (3)	0.031 (2)	0.027 (3)	−0.0056 (18)	0.002 (2)	0.0004 (19)
C43	0.028 (2)	0.026 (2)	0.028 (3)	0.0011 (16)	0.0007 (19)	0.0000 (18)
C44	0.028 (2)	0.029 (2)	0.038 (3)	0.0085 (17)	0.009 (2)	0.0056 (19)
C45	0.028 (3)	0.035 (2)	0.055 (4)	0.0027 (19)	0.010 (2)	0.009 (2)
C46	0.028 (3)	0.037 (2)	0.049 (3)	−0.0053 (19)	0.003 (2)	0.009 (2)
C47	0.028 (2)	0.027 (2)	0.029 (3)	0.0022 (17)	0.0036 (19)	0.0024 (18)
C48	0.025 (2)	0.0239 (19)	0.021 (2)	0.0026 (15)	0.0001 (18)	−0.0021 (16)
C49	0.024 (2)	0.0253 (19)	0.024 (2)	0.0007 (16)	0.0047 (18)	0.0004 (17)
C50	0.025 (2)	0.032 (2)	0.041 (3)	−0.0020 (17)	−0.004 (2)	−0.003 (2)
C51	0.024 (2)	0.026 (2)	0.027 (3)	0.0005 (16)	0.0037 (18)	0.0031 (17)
C52	0.034 (3)	0.033 (2)	0.026 (3)	0.0028 (18)	0.000 (2)	0.0022 (19)
C53	0.052 (3)	0.034 (2)	0.027 (3)	0.008 (2)	0.007 (2)	0.002 (2)
C54	0.034 (3)	0.045 (3)	0.030 (3)	0.010 (2)	0.005 (2)	0.007 (2)
C55	0.025 (2)	0.045 (3)	0.029 (3)	−0.0035 (19)	0.002 (2)	0.009 (2)
C56	0.029 (2)	0.026 (2)	0.032 (3)	−0.0077 (17)	0.001 (2)	0.0032 (18)
C57	0.046 (3)	0.053 (3)	0.043 (3)	0.023 (2)	0.004 (2)	−0.001 (2)
C58B	0.037 (4)	0.016 (3)	0.059 (5)	0.005 (3)	−0.005 (4)	−0.005 (3)
C59B	0.043 (4)	0.032 (4)	0.046 (5)	0.003 (3)	0.003 (4)	−0.006 (4)
C60B	0.045 (5)	0.042 (5)	0.053 (6)	0.012 (4)	−0.008 (5)	0.003 (4)
C60A	0.020 (5)	0.029 (5)	0.057 (8)	0.002 (4)	0.004 (5)	−0.003 (5)
C58A	0.019 (4)	0.018 (4)	0.056 (5)	−0.001 (3)	−0.006 (4)	−0.010 (4)
C59A	0.030 (6)	0.033 (5)	0.048 (6)	0.002 (4)	0.000 (5)	0.002 (5)
Br1	0.1157 (7)	0.0423 (4)	0.0534 (5)	−0.0371 (3)	−0.0287 (4)	0.0099 (3)
O1	0.035 (2)	0.0295 (18)	0.108 (4)	0.0063 (14)	0.012 (2)	0.004 (2)
O2	0.0301 (17)	0.0312 (15)	0.0257 (19)	−0.0006 (12)	0.0022 (14)	0.0020 (13)
N1	0.035 (2)	0.0274 (19)	0.034 (3)	−0.0054 (15)	−0.0088 (18)	0.0094 (16)
N2	0.038 (2)	0.0299 (19)	0.025 (2)	−0.0094 (16)	−0.0036 (18)	0.0047 (16)
C1	0.060 (4)	0.038 (3)	0.058 (4)	−0.016 (3)	−0.029 (3)	0.016 (3)
C2	0.067 (4)	0.040 (3)	0.036 (3)	−0.024 (3)	−0.019 (3)	0.011 (2)
C3	0.043 (3)	0.032 (2)	0.040 (3)	−0.008 (2)	−0.019 (2)	0.010 (2)
C4	0.030 (3)	0.030 (2)	0.082 (5)	0.0067 (19)	−0.010 (3)	0.012 (3)
C5	0.030 (3)	0.034 (3)	0.129 (7)	0.001 (2)	−0.002 (3)	0.014 (3)
C6	0.041 (3)	0.038 (3)	0.122 (7)	−0.011 (2)	−0.023 (4)	0.027 (3)
C7	0.047 (3)	0.038 (3)	0.039 (3)	−0.012 (2)	−0.009 (2)	0.010 (2)
C8	0.027 (2)	0.0239 (19)	0.026 (3)	0.0013 (16)	−0.0051 (19)	0.0023 (17)
C9	0.031 (2)	0.027 (2)	0.028 (3)	0.0014 (17)	0.005 (2)	0.0028 (18)
C10	0.025 (2)	0.034 (2)	0.041 (3)	−0.0007 (17)	−0.001 (2)	−0.005 (2)
C11	0.027 (2)	0.027 (2)	0.031 (3)	−0.0002 (16)	0.0066 (19)	0.0056 (18)
C12	0.025 (2)	0.028 (2)	0.038 (3)	−0.0060 (16)	−0.001 (2)	0.0034 (19)
C13	0.030 (2)	0.026 (2)	0.044 (3)	−0.0048 (17)	0.014 (2)	0.001 (2)
C14	0.043 (3)	0.036 (2)	0.033 (3)	0.009 (2)	0.004 (2)	0.004 (2)
C15	0.069 (4)	0.057 (3)	0.025 (3)	0.030 (3)	−0.004 (3)	0.000 (2)
C16	0.049 (3)	0.039 (3)	0.025 (3)	0.015 (2)	0.000 (2)	0.000 (2)
C17	0.065 (4)	0.051 (3)	0.043 (4)	0.028 (3)	0.008 (3)	0.008 (3)
C18	0.066 (4)	0.028 (2)	0.031 (3)	0.017 (2)	−0.017 (3)	−0.008 (2)
C19	0.110 (6)	0.031 (3)	0.026 (3)	−0.001 (3)	0.001 (3)	0.001 (2)
C20	0.077 (4)	0.035 (3)	0.070 (5)	0.024 (3)	−0.041 (4)	−0.014 (3)

### Geometric parameters (Å, º)

**Table:**

Br21—C21	1.912 (5)	C49—C50	1.531 (6)
Br41—C41	1.902 (5)	C51—C52	1.388 (6)
Br1—C1	1.907 (6)	C51—C56	1.392 (7)
O21—C24	1.357 (5)	C52—C53	1.401 (7)
O22—C28	1.235 (6)	C53—C54	1.392 (7)
O21—H21	0.8400	C54—C57	1.509 (7)
O41—C44	1.352 (6)	C54—C55	1.394 (7)
O42—C48	1.230 (5)	C55—C56	1.390 (7)
O41—H41	0.8400	C57—C58B	1.616 (12)
O1—C4	1.357 (7)	C57—C58A	1.492 (12)
O2—C8	1.224 (6)	C58A—C59A	1.469 (16)
O1—H1	0.8400	C58A—C60A	1.504 (15)
N21—C27	1.283 (6)	C58B—C60B	1.543 (13)
N21—N22	1.387 (5)	C58B—C59B	1.537 (12)
N22—C28	1.357 (6)	C42—H42	0.9500
N22—H22A	0.88 (2)	C45—H45	0.9500
N41—N42	1.391 (5)	C46—H46	0.9500
N41—C47	1.282 (6)	C47—H47	0.9500
N42—C48	1.364 (5)	C49—H49	1.0000
N42—H42A	0.882 (18)	C50—H50C	0.9800
N1—N2	1.383 (5)	C50—H50A	0.9800
N1—C7	1.284 (7)	C50—H50B	0.9800
N2—C8	1.357 (6)	C52—H52	0.9500
N2—H2A	0.88 (2)	C53—H53	0.9500
C21—C26	1.389 (7)	C55—H55	0.9500
C21—C22	1.379 (7)	C56—H56	0.9500
C22—C23	1.414 (6)	C57—H57A	0.9900
C23—C27	1.456 (7)	C57—H57D	0.9900
C23—C24	1.410 (7)	C57—H57B	0.9900
C24—C25	1.390 (7)	C57—H57C	0.9900
C25—C26	1.386 (7)	C58A—H58A	1.0000
C28—C29	1.536 (6)	C58B—H58B	1.0000
C29—C30	1.530 (7)	C59A—H59A	0.9800
C29—C31	1.532 (6)	C59A—H59C	0.9800
C31—C36	1.384 (7)	C59A—H59B	0.9800
C31—C32	1.382 (7)	C59B—H59D	0.9800
C32—C33	1.390 (7)	C59B—H59E	0.9800
C33—C34	1.395 (7)	C59B—H59F	0.9800
C34—C37	1.507 (6)	C60A—H60C	0.9800
C34—C35	1.381 (7)	C60A—H60B	0.9800
C35—C36	1.400 (7)	C60A—H60A	0.9800
C37—C38A	1.585 (9)	C60B—H60F	0.9800
C37—C38B	1.532 (10)	C60B—H60D	0.9800
C38A—C40A	1.505 (14)	C60B—H60E	0.9800
C38A—C39A	1.525 (11)	C1—C6	1.387 (10)
C38B—C39B	1.538 (13)	C1—C2	1.386 (9)
C38B—C40B	1.538 (12)	C2—C3	1.412 (7)
C22—H22	0.9500	C3—C7	1.457 (7)
C25—H25	0.9500	C3—C4	1.414 (8)
C26—H26	0.9500	C4—C5	1.390 (7)
C27—H27	0.9500	C5—C6	1.385 (9)
C29—H29	1.0000	C8—C9	1.530 (6)
C30—H30C	0.9800	C9—C11	1.539 (6)
C30—H30A	0.9800	C9—C10	1.538 (7)
C30—H30B	0.9800	C11—C12	1.393 (6)
C32—H32	0.9500	C11—C16	1.388 (7)
C33—H33	0.9500	C12—C13	1.407 (7)
C35—H35	0.9500	C13—C14	1.395 (7)
C36—H36	0.9500	C14—C17	1.517 (8)
C37—H37C	0.9900	C14—C15	1.399 (7)
C37—H37D	0.9900	C15—C16	1.389 (8)
C37—H37B	0.9900	C17—C18	1.582 (8)
C37—H37A	0.9900	C18—C20	1.522 (8)
C38A—H38A	1.0000	C18—C19	1.529 (7)
C38B—H38B	1.0000	C2—H2	0.9500
C39A—H39C	0.9800	C5—H5	0.9500
C39A—H39B	0.9800	C6—H6	0.9500
C39A—H39A	0.9800	C7—H7	0.9500
C39B—H39F	0.9800	C9—H9	1.0000
C39B—H39D	0.9800	C10—H10A	0.9800
C39B—H39E	0.9800	C10—H10B	0.9800
C40A—H40B	0.9800	C10—H10C	0.9800
C40A—H40A	0.9800	C12—H12	0.9500
C40A—H40C	0.9800	C13—H13	0.9500
C40B—H40D	0.9800	C15—H15	0.9500
C40B—H40F	0.9800	C16—H16	0.9500
C40B—H40E	0.9800	C17—H17A	0.9900
C41—C42	1.380 (7)	C17—H17B	0.9900
C41—C46	1.388 (7)	C18—H18	1.0000
C42—C43	1.405 (6)	C19—H19A	0.9800
C43—C44	1.413 (7)	C19—H19B	0.9800
C43—C47	1.464 (7)	C19—H19C	0.9800
C44—C45	1.392 (7)	C20—H20A	0.9800
C45—C46	1.385 (7)	C20—H20B	0.9800
C48—C49	1.524 (5)	C20—H20C	0.9800
C49—C51	1.543 (6)		
			
C24—O21—H21	110.00	C59A—C58A—C60A	117.7 (10)
C44—O41—H41	109.00	C57—C58A—C60A	107.6 (8)
C4—O1—H1	110.00	C59B—C58B—C60B	109.6 (7)
N22—N21—C27	117.0 (4)	C57—C58B—C60B	110.4 (7)
N21—N22—C28	118.8 (4)	C57—C58B—C59B	118.4 (7)
N21—N22—H22A	121 (3)	C41—C42—H42	120.00
C28—N22—H22A	120 (3)	C43—C42—H42	120.00
N42—N41—C47	116.0 (4)	C46—C45—H45	120.00
N41—N42—C48	118.3 (4)	C44—C45—H45	120.00
C48—N42—H42A	122 (3)	C45—C46—H46	120.00
N41—N42—H42A	118 (3)	C41—C46—H46	120.00
N2—N1—C7	116.5 (4)	C43—C47—H47	120.00
N1—N2—C8	118.9 (4)	N41—C47—H47	120.00
C8—N2—H2A	120 (3)	C48—C49—H49	109.00
N1—N2—H2A	120 (3)	C51—C49—H49	109.00
Br21—C21—C22	119.5 (4)	C50—C49—H49	109.00
C22—C21—C26	121.2 (4)	C49—C50—H50A	109.00
Br21—C21—C26	119.3 (3)	H50A—C50—H50B	109.00
C21—C22—C23	119.9 (4)	C49—C50—H50B	110.00
C24—C23—C27	122.3 (4)	C49—C50—H50C	109.00
C22—C23—C24	118.8 (4)	H50A—C50—H50C	110.00
C22—C23—C27	118.9 (4)	H50B—C50—H50C	109.00
O21—C24—C25	118.2 (4)	C53—C52—H52	120.00
O21—C24—C23	122.0 (4)	C51—C52—H52	120.00
C23—C24—C25	119.8 (4)	C54—C53—H53	119.00
C24—C25—C26	121.0 (5)	C52—C53—H53	119.00
C21—C26—C25	119.3 (4)	C54—C55—H55	119.00
N21—C27—C23	119.9 (4)	C56—C55—H55	119.00
N22—C28—C29	114.8 (4)	C55—C56—H56	120.00
O22—C28—C29	122.5 (4)	C51—C56—H56	120.00
O22—C28—N22	122.5 (4)	C54—C57—H57D	111.00
C30—C29—C31	114.2 (4)	C58B—C57—H57C	111.00
C28—C29—C30	108.8 (4)	C54—C57—H57C	110.00
C28—C29—C31	107.2 (4)	C58A—C57—H57B	108.00
C29—C31—C36	123.4 (4)	C54—C57—H57A	108.00
C29—C31—C32	118.7 (4)	C54—C57—H57B	108.00
C32—C31—C36	117.9 (4)	H57A—C57—H57B	107.00
C31—C32—C33	121.2 (4)	C58B—C57—H57D	107.00
C32—C33—C34	121.5 (5)	H57C—C57—H57D	108.00
C33—C34—C35	116.9 (4)	C58A—C57—H57A	108.00
C35—C34—C37	123.2 (4)	C59A—C58A—H58A	106.00
C33—C34—C37	119.9 (4)	C57—C58A—H58A	106.00
C34—C35—C36	121.8 (4)	C60A—C58A—H58A	106.00
C31—C36—C35	120.7 (5)	C60B—C58B—H58B	106.00
C34—C37—C38B	115.8 (5)	C59B—C58B—H58B	106.00
C34—C37—C38A	113.5 (5)	C57—C58B—H58B	106.00
C37—C38A—C39A	107.2 (6)	H59A—C59A—H59B	109.00
C37—C38A—C40A	114.3 (6)	C58A—C59A—H59C	109.00
C39A—C38A—C40A	111.6 (8)	H59A—C59A—H59C	109.00
C39B—C38B—C40B	110.2 (7)	C58A—C59A—H59A	110.00
C37—C38B—C40B	107.8 (7)	H59B—C59A—H59C	110.00
C37—C38B—C39B	114.1 (7)	C58A—C59A—H59B	109.00
C23—C22—H22	120.00	H59D—C59B—H59E	109.00
C21—C22—H22	120.00	C58B—C59B—H59D	109.00
C24—C25—H25	119.00	H59D—C59B—H59F	109.00
C26—C25—H25	120.00	C58B—C59B—H59F	109.00
C21—C26—H26	120.00	H59E—C59B—H59F	109.00
C25—C26—H26	120.00	C58B—C59B—H59E	110.00
C23—C27—H27	120.00	C58A—C60A—H60C	109.00
N21—C27—H27	120.00	H60A—C60A—H60B	109.00
C30—C29—H29	109.00	C58A—C60A—H60B	109.00
C28—C29—H29	109.00	H60B—C60A—H60C	110.00
C31—C29—H29	109.00	H60A—C60A—H60C	109.00
H30B—C30—H30C	109.00	C58A—C60A—H60A	109.00
C29—C30—H30B	109.00	C58B—C60B—H60E	109.00
C29—C30—H30A	109.00	C58B—C60B—H60F	109.00
H30A—C30—H30C	109.00	C58B—C60B—H60D	109.00
H30A—C30—H30B	109.00	H60D—C60B—H60F	110.00
C29—C30—H30C	109.00	H60E—C60B—H60F	110.00
C33—C32—H32	119.00	H60D—C60B—H60E	110.00
C31—C32—H32	119.00	Br1—C1—C2	119.7 (5)
C34—C33—H33	119.00	Br1—C1—C6	119.6 (4)
C32—C33—H33	119.00	C2—C1—C6	120.7 (5)
C36—C35—H35	119.00	C1—C2—C3	119.9 (6)
C34—C35—H35	119.00	C4—C3—C7	122.4 (5)
C35—C36—H36	120.00	C2—C3—C4	119.0 (5)
C31—C36—H36	120.00	C2—C3—C7	118.6 (5)
C34—C37—H37C	108.00	O1—C4—C3	121.6 (4)
C38A—C37—H37A	109.00	O1—C4—C5	118.9 (5)
H37A—C37—H37B	108.00	C3—C4—C5	119.5 (5)
C38B—C37—H37D	108.00	C4—C5—C6	121.0 (6)
C38A—C37—H37B	109.00	C1—C6—C5	119.8 (6)
C38B—C37—H37C	109.00	N1—C7—C3	120.1 (5)
C34—C37—H37D	108.00	O2—C8—C9	122.9 (4)
H37C—C37—H37D	107.00	O2—C8—N2	122.1 (4)
C34—C37—H37A	109.00	N2—C8—C9	114.9 (4)
C34—C37—H37B	109.00	C8—C9—C10	109.0 (4)
C39A—C38A—H38A	108.00	C8—C9—C11	106.6 (4)
C40A—C38A—H38A	108.00	C10—C9—C11	114.9 (4)
C37—C38A—H38A	108.00	C12—C11—C16	118.5 (4)
C40B—C38B—H38B	108.00	C9—C11—C16	118.8 (4)
C39B—C38B—H38B	108.00	C9—C11—C12	122.6 (4)
C37—C38B—H38B	108.00	C11—C12—C13	120.8 (4)
H39A—C39A—H39C	110.00	C12—C13—C14	120.9 (4)
C38A—C39A—H39C	109.00	C13—C14—C15	117.1 (5)
H39A—C39A—H39B	109.00	C13—C14—C17	121.5 (4)
C38A—C39A—H39B	110.00	C15—C14—C17	121.4 (5)
H39B—C39A—H39C	109.00	C14—C15—C16	122.2 (5)
C38A—C39A—H39A	109.00	C11—C16—C15	120.4 (5)
C38B—C39B—H39F	109.00	C14—C17—C18	113.9 (5)
C38B—C39B—H39D	109.00	C17—C18—C19	110.6 (4)
C38B—C39B—H39E	109.00	C17—C18—C20	109.3 (5)
H39D—C39B—H39F	109.00	C19—C18—C20	111.1 (5)
H39D—C39B—H39E	109.00	C1—C2—H2	120.00
H39E—C39B—H39F	110.00	C3—C2—H2	120.00
H40A—C40A—H40B	110.00	C4—C5—H5	120.00
H40A—C40A—H40C	110.00	C6—C5—H5	119.00
C38A—C40A—H40B	109.00	C1—C6—H6	120.00
C38A—C40A—H40A	110.00	C5—C6—H6	120.00
C38A—C40A—H40C	110.00	N1—C7—H7	120.00
H40B—C40A—H40C	109.00	C3—C7—H7	120.00
H40D—C40B—H40E	110.00	C8—C9—H9	109.00
H40D—C40B—H40F	109.00	C10—C9—H9	109.00
C38B—C40B—H40D	109.00	C11—C9—H9	109.00
C38B—C40B—H40F	110.00	C9—C10—H10A	109.00
C38B—C40B—H40E	109.00	C9—C10—H10B	109.00
H40E—C40B—H40F	109.00	C9—C10—H10C	109.00
Br41—C41—C42	120.3 (4)	H10A—C10—H10B	110.00
C42—C41—C46	121.0 (4)	H10A—C10—H10C	109.00
Br41—C41—C46	118.7 (4)	H10B—C10—H10C	109.00
C41—C42—C43	120.2 (5)	C11—C12—H12	120.00
C42—C43—C47	119.1 (4)	C13—C12—H12	120.00
C44—C43—C47	122.1 (4)	C12—C13—H13	120.00
C42—C43—C44	118.8 (4)	C14—C13—H13	120.00
C43—C44—C45	119.6 (4)	C14—C15—H15	119.00
O41—C44—C45	118.3 (4)	C16—C15—H15	119.00
O41—C44—C43	122.1 (4)	C11—C16—H16	120.00
C44—C45—C46	120.9 (5)	C15—C16—H16	120.00
C41—C46—C45	119.4 (4)	C14—C17—H17A	109.00
N41—C47—C43	119.3 (4)	C14—C17—H17B	109.00
O42—C48—C49	123.4 (4)	C18—C17—H17A	109.00
O42—C48—N42	122.1 (4)	C18—C17—H17B	109.00
N42—C48—C49	114.5 (4)	H17A—C17—H17B	108.00
C50—C49—C51	114.3 (4)	C17—C18—H18	109.00
C48—C49—C50	108.9 (4)	C19—C18—H18	109.00
C48—C49—C51	106.2 (3)	C20—C18—H18	109.00
C49—C51—C52	122.5 (4)	C18—C19—H19A	110.00
C49—C51—C56	119.0 (4)	C18—C19—H19B	109.00
C52—C51—C56	118.5 (4)	C18—C19—H19C	109.00
C51—C52—C53	120.5 (5)	H19A—C19—H19B	109.00
C52—C53—C54	121.6 (4)	H19A—C19—H19C	110.00
C53—C54—C57	122.1 (4)	H19B—C19—H19C	109.00
C55—C54—C57	121.0 (5)	C18—C20—H20A	109.00
C53—C54—C55	116.9 (4)	C18—C20—H20B	110.00
C54—C55—C56	122.1 (5)	C18—C20—H20C	110.00
C51—C56—C55	120.4 (4)	H20A—C20—H20B	109.00
C54—C57—C58A	118.9 (5)	H20A—C20—H20C	109.00
C54—C57—C58B	109.8 (5)	H20B—C20—H20C	109.00
C57—C58A—C59A	112.2 (8)		
			
C27—N21—N22—C28	165.3 (4)	O41—C44—C45—C46	178.6 (5)
N22—N21—C27—C23	178.3 (4)	C44—C45—C46—C41	0.2 (8)
N21—N22—C28—O22	−4.0 (6)	N42—C48—C49—C51	−99.8 (4)
N21—N22—C28—C29	171.9 (3)	O42—C48—C49—C50	−44.4 (5)
N42—N41—C47—C43	176.6 (4)	N42—C48—C49—C50	136.7 (4)
C47—N41—N42—C48	166.6 (4)	O42—C48—C49—C51	79.1 (5)
N41—N42—C48—O42	−9.2 (5)	C48—C49—C51—C52	−113.5 (5)
N41—N42—C48—C49	169.7 (3)	C48—C49—C51—C56	64.9 (5)
N2—N1—C7—C3	177.7 (4)	C50—C49—C51—C56	−175.0 (4)
C7—N1—N2—C8	164.5 (4)	C50—C49—C51—C52	6.6 (6)
N1—N2—C8—C9	172.0 (4)	C52—C51—C56—C55	1.5 (7)
N1—N2—C8—O2	−5.8 (6)	C49—C51—C56—C55	−177.0 (4)
C26—C21—C22—C23	−1.6 (7)	C49—C51—C52—C53	178.5 (4)
Br21—C21—C22—C23	178.3 (4)	C56—C51—C52—C53	0.1 (7)
C22—C21—C26—C25	1.1 (7)	C51—C52—C53—C54	−2.0 (8)
Br21—C21—C26—C25	−178.8 (4)	C52—C53—C54—C55	2.3 (7)
C21—C22—C23—C27	−179.8 (4)	C52—C53—C54—C57	−178.6 (5)
C21—C22—C23—C24	0.0 (7)	C53—C54—C55—C56	−0.7 (7)
C22—C23—C24—O21	−178.7 (4)	C57—C54—C55—C56	−179.8 (5)
C27—C23—C24—O21	1.1 (7)	C53—C54—C57—C58B	−104.0 (6)
C22—C23—C24—C25	2.1 (7)	C55—C54—C57—C58B	75.1 (6)
C24—C23—C27—N21	−7.4 (7)	C54—C55—C56—C51	−1.2 (8)
C27—C23—C24—C25	−178.1 (4)	C54—C57—C58B—C59B	56.4 (8)
C22—C23—C27—N21	172.4 (4)	C54—C57—C58B—C60B	−176.2 (6)
C23—C24—C25—C26	−2.6 (7)	C2—C1—C6—C5	2.0 (10)
O21—C24—C25—C26	178.2 (4)	Br1—C1—C6—C5	−175.7 (5)
C24—C25—C26—C21	1.0 (7)	Br1—C1—C2—C3	176.2 (4)
N22—C28—C29—C31	−96.7 (4)	C6—C1—C2—C3	−1.5 (9)
O22—C28—C29—C30	−44.8 (5)	C1—C2—C3—C4	−0.9 (9)
N22—C28—C29—C30	139.3 (4)	C1—C2—C3—C7	178.9 (5)
O22—C28—C29—C31	79.2 (5)	C2—C3—C4—O1	−177.4 (5)
C28—C29—C31—C36	−106.0 (5)	C2—C3—C4—C5	2.7 (8)
C30—C29—C31—C32	−167.2 (4)	C4—C3—C7—N1	−10.5 (8)
C30—C29—C31—C36	14.6 (7)	C7—C3—C4—O1	2.8 (8)
C28—C29—C31—C32	72.2 (5)	C7—C3—C4—C5	−177.0 (5)
C32—C31—C36—C35	0.3 (7)	C2—C3—C7—N1	169.8 (5)
C29—C31—C36—C35	178.6 (5)	O1—C4—C5—C6	177.9 (6)
C29—C31—C32—C33	−177.5 (5)	C3—C4—C5—C6	−2.3 (9)
C36—C31—C32—C33	0.8 (7)	C4—C5—C6—C1	−0.1 (10)
C31—C32—C33—C34	−1.0 (8)	O2—C8—C9—C10	−41.6 (6)
C32—C33—C34—C35	−0.1 (7)	O2—C8—C9—C11	82.8 (5)
C32—C33—C34—C37	−179.2 (5)	N2—C8—C9—C10	140.6 (4)
C35—C34—C37—C38B	−121.8 (6)	N2—C8—C9—C11	−95.0 (5)
C33—C34—C35—C36	1.2 (8)	C8—C9—C11—C12	−104.7 (5)
C33—C34—C37—C38B	57.3 (7)	C8—C9—C11—C16	72.0 (5)
C37—C34—C35—C36	−179.7 (5)	C10—C9—C11—C12	16.0 (7)
C34—C35—C36—C31	−1.4 (8)	C10—C9—C11—C16	−167.3 (4)
C34—C37—C38B—C40B	63.1 (7)	C9—C11—C12—C13	176.0 (4)
C34—C37—C38B—C39B	−174.2 (6)	C16—C11—C12—C13	−0.6 (7)
C42—C41—C46—C45	0.9 (8)	C9—C11—C16—C15	−174.9 (5)
Br41—C41—C42—C43	178.1 (4)	C12—C11—C16—C15	1.9 (7)
C46—C41—C42—C43	−0.8 (7)	C11—C12—C13—C14	−2.5 (7)
Br41—C41—C46—C45	−178.1 (4)	C12—C13—C14—C15	4.2 (7)
C41—C42—C43—C47	177.7 (4)	C12—C13—C14—C17	−178.2 (5)
C41—C42—C43—C44	−0.3 (7)	C13—C14—C15—C16	−2.9 (8)
C42—C43—C44—O41	−178.6 (4)	C17—C14—C15—C16	179.5 (5)
C42—C43—C44—C45	1.3 (7)	C13—C14—C17—C18	−79.1 (6)
C47—C43—C44—O41	3.5 (7)	C15—C14—C17—C18	98.4 (6)
C44—C43—C47—N41	−9.6 (7)	C14—C15—C16—C11	−0.1 (8)
C47—C43—C44—C45	−176.6 (5)	C14—C17—C18—C19	−59.9 (6)
C42—C43—C47—N41	172.5 (4)	C14—C17—C18—C20	177.5 (5)
C43—C44—C45—C46	−1.3 (8)		

### Hydrogen-bond geometry (Å, º)

Cg2, Cg3, Cg4 and Cg6 are the centroids of the C31–C36, C41–C46, C51–C56 and C11–C16 benzene rings, respectively.

**Table:**

*D*—H···*A*	*D*—H	H···*A*	*D*···*A*	*D*—H···*A*
O1—H1···N1	0.84	1.90	2.626 (5)	143
N2—H2*A*···O2^i^	0.88 (2)	1.98 (2)	2.804 (5)	156 (4)
O21—H21···N21	0.84	1.89	2.616 (5)	144
N22—H22*A*···O22^i^	0.88 (2)	1.98 (2)	2.803 (5)	155 (4)
O41—H41···N41	0.84	1.87	2.610 (5)	146
N42—H42*A*···O42^i^	0.88 (2)	1.98 (1)	2.813 (5)	158 (4)
C27—H27···O22^i^	0.95	2.55	3.218 (6)	128
C55—H55···O41^i^	0.95	2.55	3.357 (6)	142
C10—H10*A*···*Cg*4	0.98	2.78	3.734 (6)	164
C30—H30*B*···*Cg*6^ii^	0.98	2.72	3.674 (6)	163
C50—H50*A*···*Cg*2	0.98	2.78	3.733 (5)	163
C59*A*—H59*C*···*Cg*3^i^	0.98	2.94	3.824 (12)	151

Symmetry codes: (i) *x*, −*y*+1/2, *z*+1/2; (ii) *x*−1, *y*, *z*.
